# Controlling Spatio-Temporal Sequences of Neural Activity by Local Synaptic Changes

**DOI:** 10.1523/JNEUROSCI.1506-25.2026

**Published:** 2026-05-05

**Authors:** Hauke O. Wernecke, Andrew B. Lehr, Arvind Kumar

**Affiliations:** ^1^Department of Computational Science and Technology, School of Electrical Engineering and Computer Science and Digital Futures, KTH Royal Institute of Technology, Stockholm 11428, Sweden; ^2^Science for Life Laboratory, Solna 171 65, Sweden; ^3^Department of Neuro- and Sensory Physiology, University Medical Center Göttingen, Göttingen 37073, Germany

**Keywords:** computational neuroscience, dynamical networks, neuromodulation, neuroscience

## Abstract

The neural basis of behavior is believed to consist of sequential patterns of neural activity in the relevant brain regions. Behavioral flexibility also requires neural circuit mechanisms that support dynamic control of sequential activity. However, mechanisms to control and reconfigure sequential activity have received little attention. Here, we show that recurrently connected networks with heterogeneous connectivity and a smooth spatial in-degree landscape (which may arise due to asymmetric neuron morphologies) provide a robust mechanism to evoke and control sequential activity. By modulating the synaptic strength of only a few neurons in local neighborhoods, we uncovered high-impact locations that can start, stop, extend, gate, and redirect sequences. Interestingly, high-impact locations coincide with mid in-degree regions. We demonstrate that these motifs can flexibly reconfigure sequential activity, and hence, provide a framework for fast and flexible computations on behavioral timescales, while the individual parts of the pathways remain rigid and reliable.

## Significance Statement

Neuron morphologies are often asymmetric and differ in size, through which neural networks become strongly heterogeneous. Beyond being a mechanism for stabilizing network dynamics, we investigate the computational capabilities of heterogeneous networks. The resulting networks display a wide range of input connectivity across space. Hence, various cognitive processes can be computed in parallel in distinct regions of the spatial network. We demonstrate that the interactions among the computations can be flexibly reconfigured by a mechanism that utilizes local modulation. Consequently, spatially heterogeneous networks provide a framework for fast computations that can be fine-tuned in a context-dependent manner on a behavioral timescale.

## Introduction

Meaningful behavior relies on ordered sequences of thought and action (Lashley, 1951), which is reconfigured in a task- or context-dependent manner. That requires the ability of neural circuits not only to generate but also to flexibly modulate and route the activity sequences. Consistent with this idea, sequential activity has been observed in a variety of tasks such as motor control (Gallego et al., 2018; Eichenlaub et al., 2020; Lindén et al., 2022), decision-making (Harvey et al., 2012), episodic memory (O’Keefe, 1976; Dragoi and Buzsáki, 2006; Foster and Wilson, 2006; Diba and Buzsáki, 2007; Pastalkova et al., 2008; Buzsáki and Tingley, 2018), olfactory processing (Friedrich and Laurent, 2001), and birdsong generation (Hahnloser et al., 2002).

The emergence of sequences requires the formation of feedforward pathways (functional or anatomical) in a recurrent network. Attractor dynamics combined with spike frequency adaptation or synaptic depression, supervised learning (i.e., echo-state networks), unsupervised learning, and even finite size effects can all facilitate the formation of feedforward networks (Zhang, 1996; Vogels and Abbott, 2005; Rabinovich et al., 2006; Rokni and Sompolinsky, 2012; Rajan et al., 2016; Lindén et al., 2022). These mechanisms assume that the (initial) network connectivity is homogeneous and “symmetry of connectivity” has to be broken by dynamics, neuron properties, or learning. Neuron morphology alone can break the symmetry of connectivity and facilitate the emergence of sequential activity. Axonal projections and dendritic branches extend out asymmetrically in a preferred direction (Jiang et al., 2015; Mohan et al., 2015; Narayanan et al., 2015; Xu et al., 2016; Peng et al., 2021; Weiler et al., 2022; Kurth et al., 2025). Furthermore, recent data suggest that in the mouse sensory cortex, nearby neurons project preferentially in similar directions, aligned with the propagation of traveling waves of activity (Ye et al., 2023). Network models with such features of asymmetric neuron morphologies naturally have feedforward pathways (Spreizer et al., 2019).

In contrast to the mechanisms underlying the emergence of sequences, mechanisms to control and reconfigure sequential activity have received little attention. Synaptic plasticity would be too slow to reconfigure interactions among neural sequences on behavioral timescales. Mechanisms based on attractors with adaptation (in synapses or neurons) would rely on continuous external steering (Kurikawa and Kaneko, 2021). Thus, the problem of flexible control of neural activity sequences on behavioral timescales remains poorly understood.

**Figure 1.**
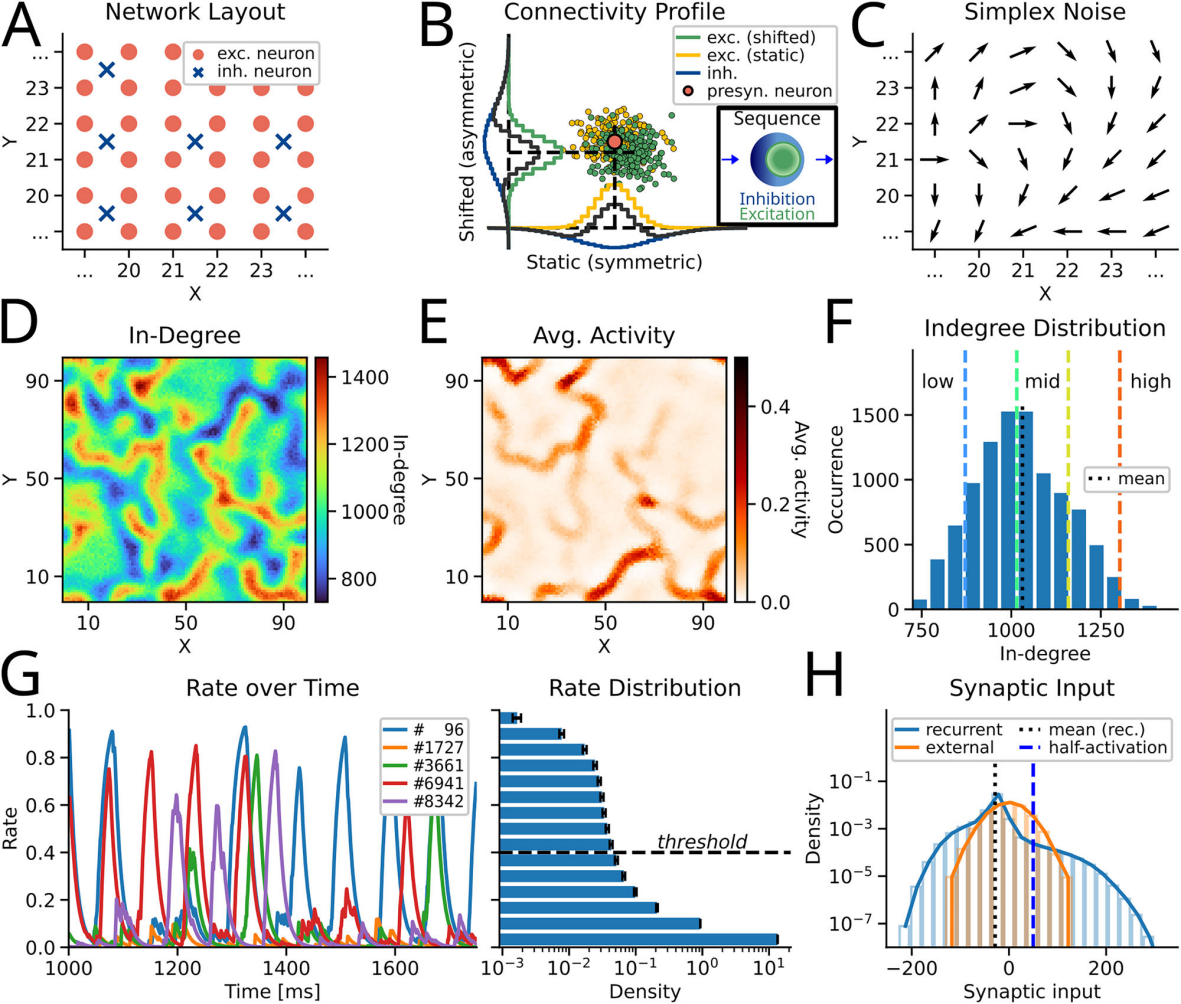
Network architecture and dynamics. ***A***, Network layout: red dots represent the location of excitatory neurons and blue crosses those of inhibitory neurons. The network has a toroidal structure to avoid boundary effects. ***B***, Connectivity kernel: The pre-synaptic neuron (red dot) shifts its targets (yellow) toward a preferred direction *ϕ* (here: bottom right), resulting in new post-synaptic targets (green). A histogram of the targets resembles then an asymmetric Mexican-hat distribution (*y*-axis; symmetric case on the *x*-axis; blue represents the inhibitory connectivity kernel, gray the sum of excitatory and inhibitory kernels). Inset: Sequence schematic with an excitatory center (green) and an inhibitory surround (blue) traversing from left to right (arrows). This asymmetry forms the basis of moving sequential activity, and is trailed by an inhibitory “shadow.” ***C***, Schematic of preferred directions *ϕ*’s which were chosen using a gradient noise algorithm (see Methods). ***D***, The in-degree of the excitatory neurons reveals high and low in-degree regions. ***E***, Average activation of the excitatory neurons: The output rate of each neuron was averaged across time. As expected, higher activation occurs on regions with high in-degree (cf., panel ***D***). ***F***, Distribution of in-degrees across five networks: black dashed line indicated the mean. The dashed colored lines separate low, low-mid, mid, mid-high, and high in-degree regions (for more details see Methods). ***G***, Left panel: The activity rate of several neurons across time. Right panel: Histogram of the activity rates of all neurons across time. Black error bars indicate the standard deviation across baseline simulations (*S*_seeds_ = 8). ***H***, Histogram of the external input (orange) and the recurrent synaptic input (blue) across all neurons and across time. The network is inhibition stabilized as the mean recurrent (black dashed line) input is lower than zero. The blue dashed line indicates the half-activation point of the sigmoidal activation function.

The model proposed by Spreizer et al. (2019) appears too hardwired to suggest any mechanism for controlling neural activity sequences. The model is based on a directional asymmetric connectivity kernel, in conjunction that adjacent neurons have similar preferential directions. Together, they form heterogeneous networks with regions of low, mid, and high in-degree (Fig. 1). This degree heterogeneity gives rise to circuit motifs that can be used to control sequential dynamics.

We show that in such networks, even small changes in the local connectivity can introduce large changes in the dynamics and interaction among sequences. Somewhat counterintuitively, a subset of locations with mid-range in-degree has the biggest impact on the sequence dynamics. We systematically uncover motifs where small modulation of local connectivity can modulate the neural activity, i.e., start, stop, extend, gate, and select sequences. In our model, motifs are found in medium in-degree regions that act as “switches” of different kinds to rapidly reconfigure the trajectory of sequences. Our work provides insights into how the spatial geometry of network connectivity could be exploited for computations, as the existence of these motifs depends on the locally correlated connectivity of neurons.

Neuromodulators (NMs) are released in a patchy manner (Patriarchi et al., 2018; Sun et al., 2018; Glaeser-Khan et al., 2024), and can modulate neural excitability and synaptic strength on a behavioral timescale (Nadim and Bucher, 2014; Colangelo et al., 2019). Our results provide a putative mechanism by which neuromodulation changes network dynamics qualitatively by reconfiguring sequential activity, thereby contributing to behavioral flexibility and learning new skills.

## Material and Methods

### Neuron model

Here, each neuron was modeled as a firing rate-based unit. The firing rate dynamics were modeled as:

$$\tau_{M}\frac{dr}{dt}=-r+\Phi (I(t))\;\text{with}\;(1)$$

$$\Phi (I(t))=\frac{1}{1+e^{\beta (I_{0}-I(t))}},\;(2)$$

where *τ*_M_ is the membrane time constant. *β* determines the steepness of the transfer function, and *I*_0_ defines the external input that results in an output of *r*_0_ = 0.5 in the absence of any network connectivity.

The total input to a post-synaptic neuron is defined as

$$I_{\mathrm{post}}(t)=\sum_{\mathrm{pre}=1}^{N}w_{\;\mathrm{post},\mathrm{pre}}\;*\;r_{\mathrm{pre}}(t)-I_{\mathrm{ext}}(t).\;(3)$$

The weights *w* and the external input *I*_ext_ will be discussed in detail later. All parameters of the network and neurons are given in Table 1.

**Table 1.** Parameter of the network and its neurons

Parameter	Value
nrows	100
No. of exc. neurons	10,000
No. of inh. neurons	2,500
*I*_0_	50
*β*	0.25
*τ*_M_	12 ms
*J*	0.275
*g*	8
*μ*_ext_	0
*σ*_ext_	30
*t*_sim_	4,000 ms
*t*_warmup_	400 ms
*δt*	1 ms
*σ*_exc_	2.5
*σ*_inh_	4.5

### Network model

The network consisted of 10,000 excitatory neurons and 2,500 inhibitory neurons, called the excitatory and inhibitory population, respectively. This resembles a ratio of 4:1 as found in the mouse neocortex (Braitenberg and Schüz, 1998). The neurons were placed on a 100-by-100 grid with equal spacing. The boundaries of the grid form a torus. This was done to avoid boundary effects. The inhibitory neurons were placed at the center of four excitatory neurons, ensuring equidistance among them (Fig. 1*A*).

Each neuron formed 3,750 (938) connections to the excitatory (inhibitory) population. Multiple connections were allowed, while self-connections were prohibited. The connection probability followed a Gaussian distribution, with different spreads *σ*_*p*_ for the excitatory and the inhibitory population,

$$\begin{array}{rl} \;f(X) & =f(X_{1},X_{2})\\ & ={\left(\frac{1}{2\pi }\right)}^{\;p/2}|\Sigma {|}^{-1/2}\exp\;\left[-\frac{1}{2}(X-m{)}^{\prime }\Sigma^{-1}(X-m)\right]. \end{array}\;(4)$$

In this 2D case with no correlation among the directions, the width of the distributions was

$$\Sigma =\left[\begin{array}{cc} \sigma_{p}^{2} & 0\\ 0 & \sigma_{p}^{2} \end{array}\right],\;(5)$$

with p∈{exc,inh} for the excitatory and inhibitory population, respectively. The higher spatial spread of the inhibitory population resulted in an effectively Mexican-hat connectivity kernel with local excitation but long-range inhibition (Fig. 1*A*).

The overall connection strength *w*_post,pre_ between the *pre*-synaptic and the *post*-synaptic was calculates as

$$w_{\mathrm{post},\mathrm{pre}}=k_{\mathrm{post},\mathrm{pre}}J\;\left(\text{exc.}\right)\;\text{and}\;(6)$$

$$w_{\mathrm{post},\mathrm{pre}}=-k_{\mathrm{post},\mathrm{pre}}gJ\;(\text{inh.}),\;(7)$$

with *k*_post,pre_ being the number of formed connections, *J* was taken as the synaptic weight, and *g* the ratio of inhibitory and excitatory synapses.

#### Simplex noise and in-degree landscapes

As Spreizer et al. (2019) showed, spatio-temporal activity sequences (STAS) arise when (1) individual neurons preferentially make some of their connections in a specific direction *ϕ*, and (2) the *ϕ*’s of neighboring neurons are similar. The spatial correlations in the network are based on a 2D Simplex noise—a class of gradient noise (Perlin, 2001; Duncan, 2018). Conceptually, random vectors are instantiated on a 2D grid, and a set of four grid points form a cell. Then, the value of a test point within a cell is calculated based on the dot-product between the random vectors of the grid and the vectors pointing from the grid points toward the test point. Given a second test point in the vicinity, its value will be similar to the vectors pointing to this test point as before. Consequently, the values within a cell are changing smoothly across space, still depending on random vector instantiations—hence the name gradient noise. An important parameter is the sample frequency of points within a cell, as it controls the extent of spatial correlations. In our case, using only 4 grid points, then the preferred directions of the neurons are highly correlated. On the other side of the spectrum, a grid of 100-by-100 random vectors would assign a random preferred direction to each neuron (cf., Perlin size in Spreizer et al., 2019). Furthermore, the spatial correlation relates loosely to the width of a pathway, hence a grid size is chosen so that several pathways exist across the network.

#### External input

Additional external current was modeled as an approximation of Gaussian white noise (GWN), with mean *μ*_ext_ and variance *σ*_ext_. The external input was sampled independently for each neuron.

#### Network simulation

The network activity was simulated for *t*_sim_ = 4,000 ms, with a preceding warm-up phase (*t*_warmup_ = 400 ms). As different sources of stochasticity exist in the model, we ran the simulations re-sampling the external noise input each time. For simplicity, the connectivity was fixed across the implementations of the GWN. We tested that the dynamics were robust against randomness in the connectivity. Thus, we defined the baseline activity across a connectivity landscape for the simulation time *t*_sim_, and with an implementation/seed of the external noise leading to *S*_seeds_ = 8 baseline simulation.

The network was simulated using the brian2 framework (Stimberg et al., 2019).

#### In-degree and its domains

The in-degree was calculated as the sum of excitatory-to-excitatory connections weighted by the synaptic weight *J*. The inhibitory-to-excitatory connections were not considered as those projections are isotropic and symmetric, and hence, only acts as a offset.

The in-degree was divided into five domains: low, low-mid, mid, mid-high, and high in-degree domains. The domains were equally distributed between the lowest and the highest in-degree. Although that resulted in an unbalanced amount of neurons per domain, it prevented thinner domains at which we observed many in-degree, mostly the mid in-degree domain.

As the patches covered many neurons with a potentially wide range of in-degrees, splitting domains from the lowest to the highest in-degree would result in zero patches in the low, or the high in-degree region. Therefore, the lower and upper limits were adjusted to the 2.5th and the 97.5th percentile respectively.

### Neuromodulation

To modulate the sequence dynamics, we defined a small local neighborhood called a patch, with a center *c*, and radius *r* (*r* = 6 grid points). From each patch, we randomly chose *N*_rec_ = 40 neurons. For these neurons, all incoming synapses were modulated—i.e., the weights were either increased or decreased by p=±10%. This kind of modulation of local connectivity is inspired by the way NMs act in the brain (Nadim and Bucher, 2014; Patriarchi et al., 2018). Therefore, with the assumption that NMs often act on a longer timescale than neural activity, the patch was active throughout a simulation, i.e., the connection strength remained higher or lower through a specific run of the network.

### Detection of sequences

#### Toroidal density-based spatial clustering of applications with noise algorithm (DBSCAN)

A sequential activation of neurons across space and time was detected as sequences following a multi-step algorithm. First, the rate of each neuron was translated into pseudo-spikes by thresholding (Θ = 0.4). These spikes were then clustered using the DBSCAN (Ester et al., 1996) to remove pseudo-spikes that were not participating in any sequence ( ϵ=4, min_samples = 50, Pedregosa et al., 2011). Note that the dimensions of the data were in *x*-, *y*-directions, and across time. Although re-scaling of the time axis could change the relation of space in time, it appeared not to have a strong effect.

As the network space was a torus, the standard DBSCAN cannot account for activity that convolves around the edges. To account for this, the activity was shifted by *nrows*/2 in *x*- and *y*-directions, and the DBSCAN clustered the pseudo-spikes again. The resulting clusters were then merged. Further on, each cluster represents a sequence. As spatio-temporal sequences are of interest, we removed very short sequences that did not traverse at least 8 grid points.

#### Sequence detection

In each sequence, many neurons were active. Here, we count the number of sequences each neuron participated in. That resulted in the sequence landscape. When investigating how many sequences crossed a certain location or pathway, we incorporated neurons within detection spots (circle with radius *r* = 2). Then, we counted the number of sequences in which at least one neuron from within the detection spot participated in. If different subsets of neurons were active across different sequences, this approach removed this uncertainty.

The second feature, the duration of a sequence was derived from the first and last time points at which the sequence was registered.

#### Network-wide effects of modulation

The network-wide effects of modulation are shown in two different ways: (1) the mean of the sequence count and the average duration with its standard error of the mean across different instances of external drive is depicted for each patch/set of neurons individually and (2) a density-based visualization in which the effects of all patches/sets of neurons are analyzed using a Gaussian kernel. The limits of the resulting probability density function (pdf) were then chosen to match the outlines of the individual effects.

### Active neurons during competition and cooperation

To determine the number of active neurons during competition and cooperation, we identified sequences that traversed the detection spots of the branches *M*, *B*_1_, and *B*_2_ within a time window of *t*_window_ = 200 ms. Then, the sequence (sequences in case of competition) was binned along the time axis resulting in the number of active neurons per time point.

### Max-tree algorithm

A max-tree algorithm represents an image via connected components that were obtained by various thresholds (Salembier et al., 1998; Souza et al., 2016). Thresholding a grayscale image results in a binary image, then the *white* pixels form components. When the threshold is smaller, these components become connected and merge.

In our case, regions with a high sequence count formed the initial components, lowering the threshold resulted in joint components. At this threshold, the neurons that shaped the bridge were considered semi-transmissive. Together, they formed the unreliable pathway between two reliable pathways. Repeating this merging process until the threshold of zero resulted in a merge tree that linked all reliable pathways. As a merging point is related to the transmission probability between two reliable pathways, changing the probability appeared to control mechanism for routing sequences across the network. Consequently, the amount of merges suggested the lower limit of such control sites. Finally, the number of individual end-nodes indicate how many separated pathways exists.

This naive identification of semi-transmissive neurons contained artifacts that contained neurons that were far from one of the components/pathways. We added a filter that required bridge neurons to be in the vicinity of both components (maximum distance to both components *d*_max_ ≤ 4). Moreover, we included all neurons that were neighbors of such bridge neurons if they were not further away from both components than 10 grid points.

### Task-specific network

We utilized the same network parameters (e.g., *τ*_M_ and all the others, cf., Table 1) as for the previous network. However, the preferred directions *ϕ* of the neurons were customized to realize the task structure. The Gate and Select motif were designed symmetrically, but the randomness of the connectivity can induce imbalance. Neurons that were far from the task-relevant structure did not have a preferred direction. The detection spots S, M1, and M2 were chosen to be between two motifs, i.e., between start-gate, gate-repeat, and repeat-select, respectively. The detection spots L and R were the read-out spots after the select motif.

## Results

We are interested in understanding how STAS in neuronal networks can be modified or controlled. To investigate this, we used the model proposed by Spreizer et al. (2019) to generate STAS. For the control of STAS dynamics, we studied the effect of changing a small fraction of synapses in a small neighborhood mimicking local release of a NM, which could enhance or reduce synaptic strength, and characterized the lifetime and number of unique STAS.

### Model of spatio-temporal sequences

Typically, in networks with distance-dependent connectivity, neurons connect in all directions with equal probability. When the spatial extent of inhibitory neurons exceeds that of excitatory neurons, the network dynamics exhibits a multiple bump solution (Amari, 1977; Gozel and Doiron, 2024). Spreizer et al. (2019) showed that spatio-temporal sequences of neural activity can emerge when either excitatory or inhibitory neurons project in a spatially asymmetric manner, such that 2–5% of their targets lie in a preferential direction *ϕ*, and *ϕ*’s of neighboring neurons are similar (Fig. 1*C*). Given that neighboring neurons project in a similar direction, activity is transmitted toward that direction. Consequently, the multiple bump solution becomes unstable, and STAS emerge. This modification in the connectivity rule results in an asymmetric Mexican-hat-type connectivity kernel (Fig. 1*B*).

Here we implemented the connectivity rule proposed by Spreizer et al. (2019) in a spatial network where each neuron was modeled as a firing rate-based model (see Methods, Fig. 1*A*–*H*). Spreizer et al. (2019) argued that when neurons had a preferred direction for their projections, they formed anatomical chains that composed the basis of temporal sequences in the network. Moreover, the spatial correlation among preferred directions imposed constraints on the number and length of sequences. Besides this, we also found that even though all neurons had the same out-degree, the in-degree of neurons showed a wide distribution and a spatial structure (Fig. 1*D*,*F*; Fig. S1). Because in our model inhibitory neurons projected in an isotropic manner, this spatial landscape of excitatory in-degree directly implied a landscape of high and low excitation–inhibition (EI) ratios.

It has already been described that structural variability in the connectivity can lead to an inhomogeneous distribution of EI ratios (Landau et al., 2016). Here we show that the variable EI ratio may also have a spatial structure. In our network, EI ratio variability did not lead to network-level instabilities nor saturation of rates (Fig. 1*E*,*G*); therefore, we did not seek to balance the individual neurons. Instead, we explore how the spatial distribution of EI ratios can be leveraged to control the STAS dynamics.

Throughout a simulation, we injected uncorrelated GWN to all the neurons to initiate the dynamics (Fig. 1*H*). STAS dynamics unfolded stochastically in regions of high and mid in-degree (red and green regions), which we refer to as “pathways.” These pathways formed along neurons with aligned preferred directions *ϕ* (Fig. 1*D*,*E*). Several STAS traversed the network simultaneously (Movie 1; Fig. S2). Given the asymmetric connectivity of excitatory neurons, traveling excitatory activity was accompanied by an inhibitory “shadow” (inset in Fig. 1*B*). This inhibitory shadow formed the basis of interaction among STAS. Due to the long-range inhibition, multiple STAS might compete along the same pathway, or even across neighboring pathways. Overall, multiple factors like the input drive, the connectivity, as well as the spatial structure of the preferred projection directions of the neurons (*ϕ*) could affect the network dynamics.

**Movie 1.** Neural activity over time. Sequences arose in various regions, were active simultaneously, and eventually extinguished across the network. [View online]

### Effect of local change in synaptic connectivity on sequence dynamics

As the activity unfolded along high in-degree regions, the spatial structure of the EI ratios suggested that network response to a spatial external stimulus or transient perturbation should depend on its location (Fig. 1*D*,*E*). Perhaps more interestingly, the spatial inhomogeneities in the EI balance also suggested that the effect of changing synaptic weights in local patches of the network would have a bigger effect on the network activity than non-local changes. That is, changes in synaptic weights within a local neighborhood (as might happen during local release of NMs) could be used as a way to control or shape the dynamics of STAS in a task- or context-dependent manner.

To illustrate and characterize this effect, we randomly selected 40 (out of 113) excitatory neurons from a circular region, hereafter referred to as patches, with a radius of 6 grid points (or neurons). For these neurons, the strengths of incoming synapses were then changed by ±10%. We quantified how such a modulation affects the spatio-temporal dynamics in the network. To this end, we thresholded the neural activity and obtained pseudo-spikes which were then clustered using DBSCAN. Each cluster was considered a STAS (see Methods). The total number of STAS and their average duration were analyzed. The baseline was obtained without any modulation of the connectivity matrix.

**Figure 2.**
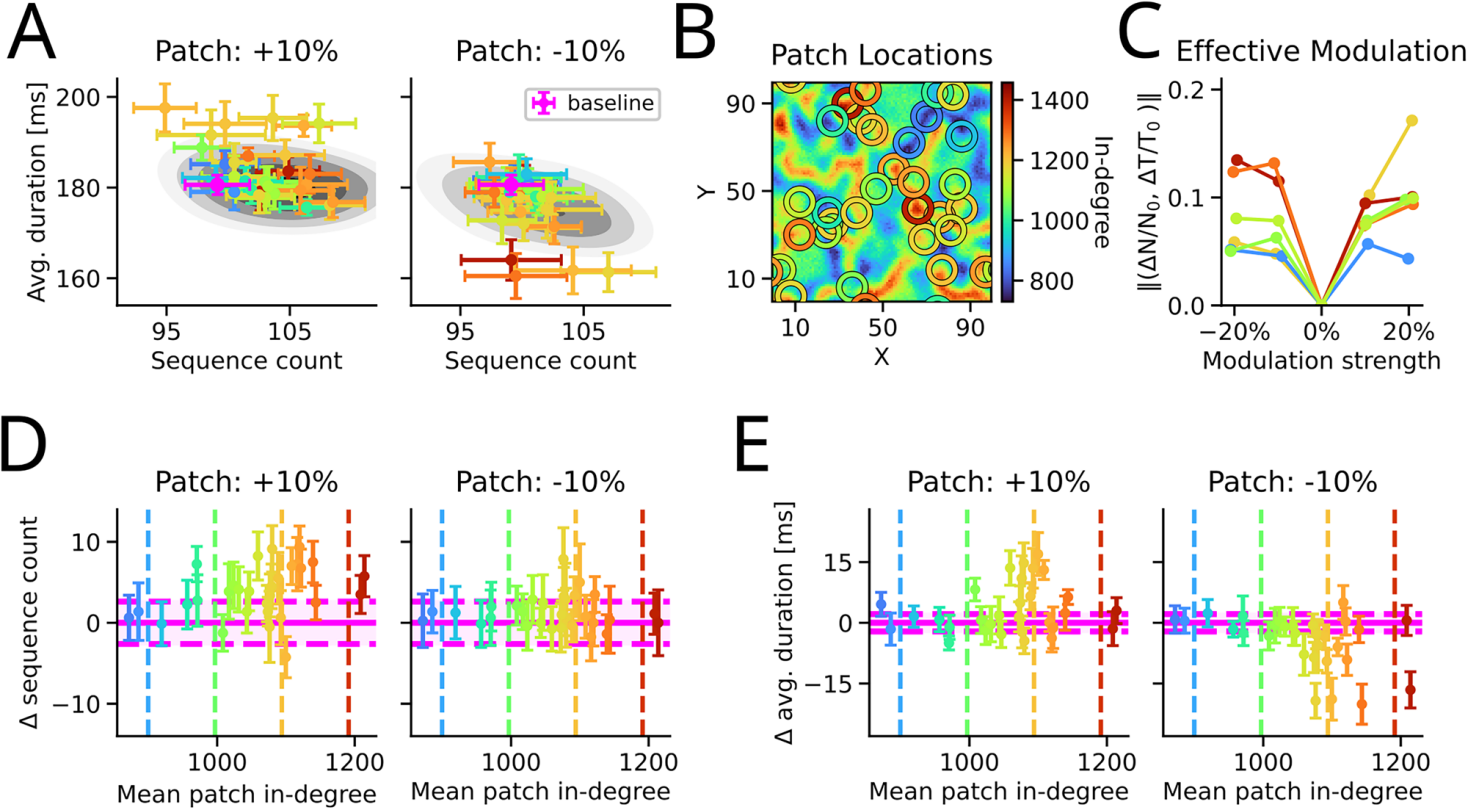
Effect of local and non-local changes in synaptic connectivity on the number of sequences and their duration. All panels: baseline simulations are performed without modulation of the connectivity. ***A***, Random spatial patch locations: in each location neurons from a small neighborhood were chosen and their connectivity was either increased or decreased by 10%. Such a modulation of connectivity resulted in large changes in the number and/or duration of STAS [error bars are the sample standard error of the mean (SEM) across random seeds of input noise]. Here, results from 30 randomly chosen locations are shown. In comparison, modulation of 40 random neurons exhibited a smaller effect (gray contour; Fig. S3). Color indicate the average in-degree of the patch (cf., ***D*** and ***E*** for reference). ***B***, Patch locations: the random locations of ***A*** are depicted on the in-degree landscape (colored circles). The color indicates the mean patch in-degree of the location. ***C***, Effect of the modulation strength: a change in modulation strength is non-linear and non-monotonic. Moreover, the effect of the modulation highly depends on the sign of the modulation. ***D***, Difference in sequence count of STAS compared to baseline over the mean in-degree of the patch location. The shaded area indicate the SEM across the baseline simulations. Locations as in ***A***. Error bars are the SEM across simulations. ***E***, Same as in the panel ***C***, but the difference in average duration.

First, we increased the incoming excitatory-to-excitatory synapses of as few as 40 neurons (only 0.32% of the entire population) in random patch locations (Fig. 2*B*). We observed that such modulation often increased the average lifetime of sequences while also tending to increase the sequence count (Fig. 2*A*, left panel).

Interestingly, when we decreased the synaptic strength of synapses of a local group of neurons, the sequence count was stable or slightly increased, while the average duration tended to drop (Fig. 2*A*, right panel). It is worth mentioning that some landscapes evoked a local stationary activation bump besides sequential activity in the remaining network. Such stationary bumps arose due to very high convergence, which led to saturation in the excitatory rates. With such high activity recurrent connectivity was sufficient to elicit persistent activity. Similarly, a (positive) modulatory patch rarely lead to the formation of such static activity bumps. We excluded those networks and patches from our analysis.

As a control, we also tested the effect of increasing or decreasing the strength of incoming synapses of 40 randomly chosen neurons from throughout the network. A comparison of patchy versus non-spatial modulation should reveal the collaborative effect that arises due to the shared connectivity. Such an effect would be missing when neurons are chosen randomly. Indeed, non-spatial modulation did not strongly alter the STAS count and duration (gray contour in Fig. 2*A*; Fig. S3).

Overall, spatially localized changes in synaptic strengths resulted in a wider range of responses than non-spatial modulation. Part of this variance can be explained by the modulation sign and by the location or the in-degree of the stimulated neurons (Fig. 2*C–E*). The strongest effects were observed when the synapses were altered in mid and mid-to-high in-degree regions. However, not all mid and mid-to-high in-degree regions had strong impact. Positive modulation seemed to be potent in increasing the sequence count over a large range of in-degrees, and effective in prolonging sequences when mid-to-high in-degree regions were modulated.

Weakening of synapses in a patch modulated the activity strongest in mid and mid-to-high in-degree regions (Fig. 2*A*,*D*,*E*, right panels). The primary effect was to shorten the duration of sequences, and to exhibit more more sequences in the network. These two effects may be entangled as shorter sequences entail less competition, which in turn would allow more sequences to form. Although the effect of the patch modulation was measured by the network dynamics, major deviation in STAS features (count and duration) points toward a local mechanism that reshapes network dynamics. Hence, we further focused on locations which could reshape the sequence dynamics locally.

### Semi-transmissive neurons

In our rate model, neurons’ maximum sensitivity to the input is by definition around the half-activation point of the sigmoidal transfer function. Therefore, intuitively, controlling the output by small changes in the input requires that the input to the neurons keeps them close to the half-activation point of their transfer function. That is, for maximal sensitivity a neuron should operate in a medium transmission regime. Very strong connections lead to saturation and only strong weakening displays an effect. On the other hand, very weak connections needs to be greatly facilitated to evoke a post-synaptic response (Fig. S4).

**Figure 3.**
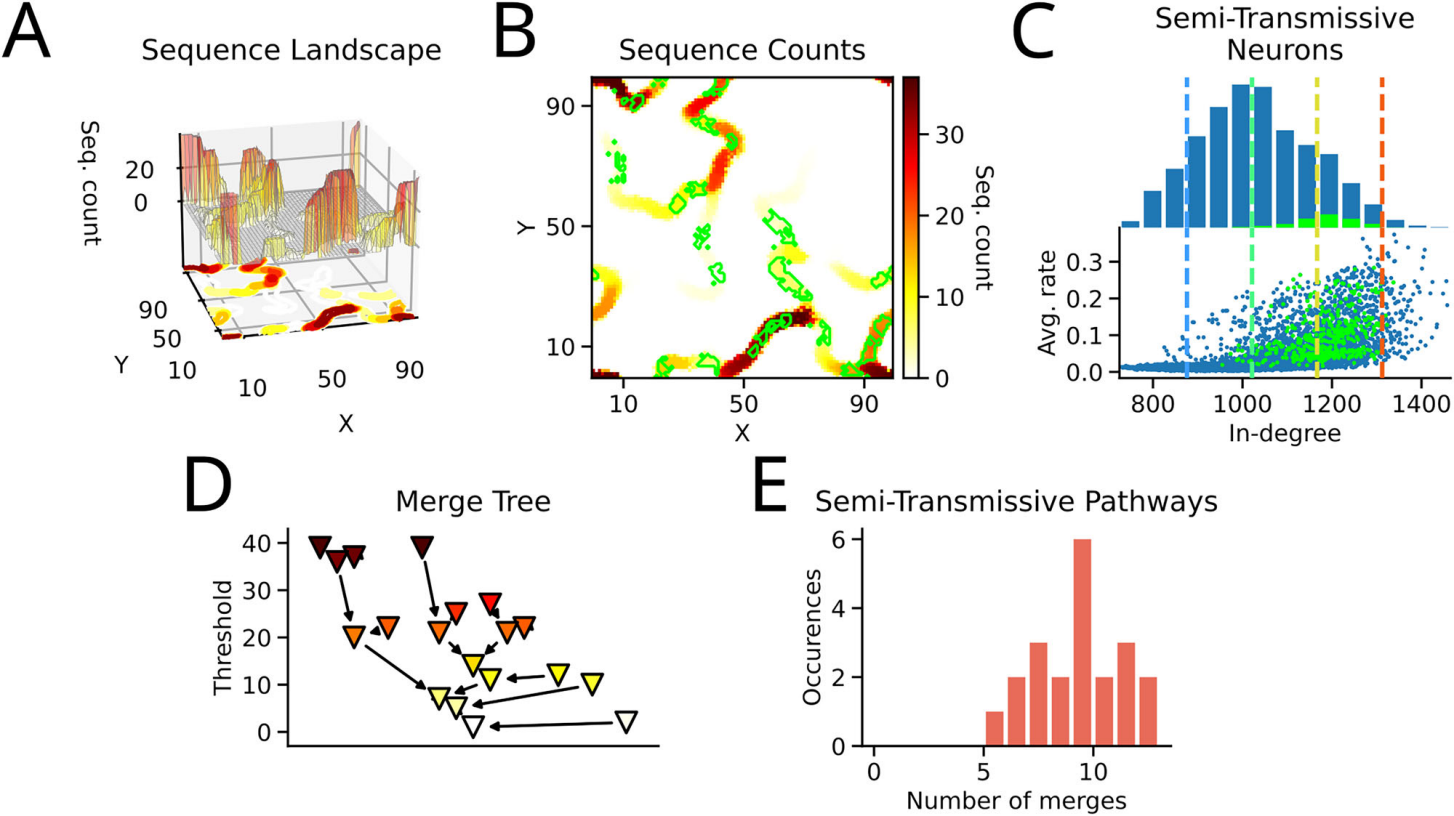
Semi-transmissive neurons populate mid and mid-to-high in-degree regions. ***A***, Sequence landscape: the number of identified sequences across the network formed a landscape which was used to identify unreliable pathways. ***B***, Sequence counts on location. Color indicates the number of sequences in which a neuron participated in (see Methods). Green regions indicate semi-transmissive neurons. ***C***, Top panel: Histogram of in-degree (cf., Fig. 1*F*) featuring the in-degree of semi-transmissive neurons (green); Bottom panel: Avg. rate of all neurons over in-degree (semi-transmissive neurons are indicated in green). ***D***, Exemplary merge tree: Two reliable pathways merge together via an unreliable pathway at a lower threshold (of the sequence count). Colors as in ***B***. ***E***, Histogram of merges per tree across networks: Each merge tree has *m* merges, i.e., *m* unreliable pathways. Here, the histogram of detected merges in the baseline simulations across 20 different networks is shown.

In our model, a reliable pathway is characterized by perfect transmission. In contrast, an unreliable pathway transmits sequences probabilistically, i.e., a fraction but not all sequences are transmitted. As an unreliable pathway can be modulated to increase or decrease the transmission probability, the leverage for controlling sequences via a local mechanism is high. We identified regions of unreliable transmission using a merge tree analysis (see Methods). In brief, a threshold was lowered and distinct peaks in the (sequence) landscape emerged. Lowering the threshold further eventually merged the peaks. Analogously, two distinct reliable pathways were linked via an unreliable pathway characterized by a lower sequence count (Fig. 3*A*; see Methods). Neurons that bridged two reliable pathways were found across all pathways (Fig. 3*B*, green regions). These semi-transmissive neurons were non-specific to a range of average rate. But interestingly, they were predominantly found in regions of mid and mid-to-high in-degree (Fig. 3*C*).

Not only were semi-transmissive neurons identified, but also the number of merges indicated how many locations were potentially well-suited to route sequences through the network. Across 20 different networks, we discovered ≈9 such locations on average. As transmission probability might depend on the external drive, this number of locations was a lower limit since more unreliable transmissions could arise in longer simulations or across different instances of external drive (Fig. 3*D*,*E*). Together with the previous results, we further investigated the characteristics of high-impact locations and their transmission probability to identify network motifs which modulate the sequences systematically.

### Motifs underlying the effect of local synaptic changes on STAS dynamics

The observation that a subset of mid and mid-to-high in-degree regions were particularly susceptible to perturbations as well as being characteristic for unreliable pathways suggested that local synaptic/neural changes might be a mechanism to control STAS dynamics. There are multiple ways in which these local perturbations may arise, for example, from the spatially localized release of NMs (Patriarchi et al., 2018; Sun et al., 2018; Glaeser-Khan et al., 2024), sites of patchy synaptic connections (Lund et al., 2003), or even volume transmission (Liu et al., 2021). In our model, given the geometry of the connectivity, mid in-degree regions could be surrounded by high- and/or low in-degree regions, and that gave rise to specific motifs enabling control over STAS dynamics.

#### Starter

**Figure 4.**
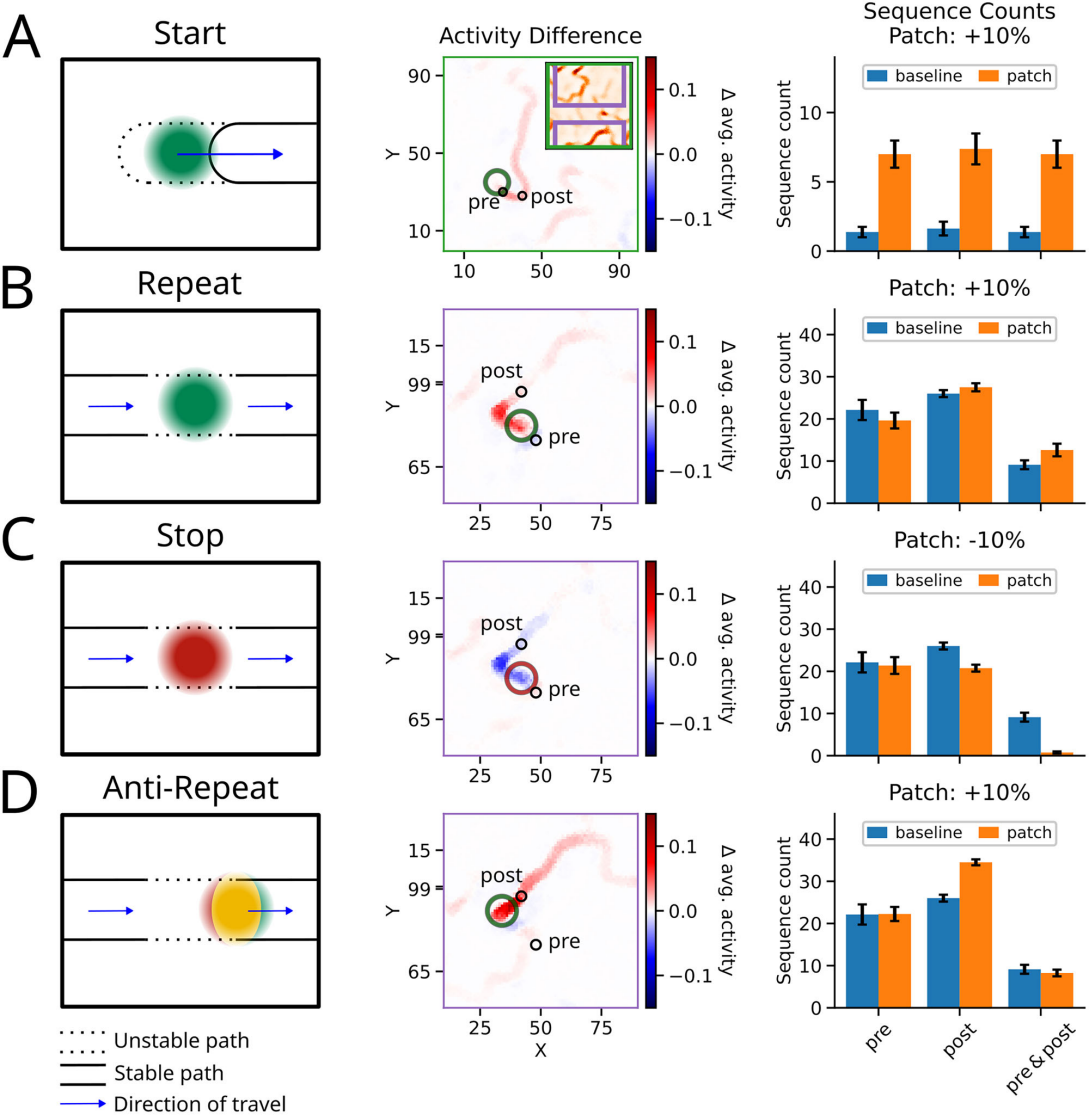
Effect of changes in the local connectivity within a single pathway. Each motif was identified in the network described in Figure 1. ***A***, Left panel: schematic of a Start motif. The local patch (colored circle) was at the beginning of a pathway to evoke more sequences and to prepend the pathway. Solid lines represent a pathway with reliable transmission; dashed lines represent an unreliable region at which transmission is stochastic (see legend at the bottom). Middle panel: an example of a Start motif in the network. The colors show the change of activity to the baseline imposed by the Start motif. The motif location is indicated with a large circle; cf., Figures 1*E* and 3*A*,*B*. For visual clarity we focused the network to bring the motif in the middle of the plot. This is indicated by the inset. Right panel: number of sequences for detection spots *pre*, *post*, and subsequent activation of *pre & post* (detection spots are indicated by small black circles in the middle panel; see Methods). Error bars indicate SEM across simulations (*S*_seeds_ = 8). ***B***, Same as in ***A***, but for the Repeat motif: an increase in connectivity of neurons in this location raised the probability of sequence transmission. ***C***, Same as in ***A***, but for the Stop motif: A decrease in connectivity of neurons in this location decreased the probability of sequence transmission. ***D***, Same as in ***A***, but for the Anti-Repeat motif: an increase in the connectivity of neurons in this region triggered the independent emergence of STAS. Such new and spurious STASs competed and could suppress other pre-existing sequences.

We refer to the mid-degree regions at the start of a pathway as a Start motif (Fig. 4*A*, left panel) because a change in the local connectivity in these regions can affect the activity in two ways: (1) to initiate sequences with a higher probability and (2) to prepend the pathway increasing its overall range.

To illustrate the effect of Starter locations, we used the same network as shown in Figure 1*D* (and its average activity in *E*). We identified a Starter location (Fig. 4*A*, middle panel, green circle), and studied its effect on the sequence count. For the quantification of local changes in sequence count, we registered all STAS that crossed small spots along the pathway (Fig. 4*A*, middle panel, black circles, for details see Methods).

We found that the Starter patch increased the number of sequences locally, i.e., the spot marked as *pre* (by 510% compared to baseline; Fig. 4*A*, right panel). Given an activation at *pre*, the Starter patch retained perfect transmission of its activity to the pathway, i.e., the *post* spot [*pre* → *post*: *P*(*post*|*pre*): 100% (patch and baseline)]. Furthermore, in the presence of the Starter patch, if there was an activity at *post*, it was more likely to have originated at *pre* [i.e., *P*(*pre*|*post*): 95% (patch) vs 85% (baseline)]. Thus, the Starter patch increased the transmission along a pathway and also reduced the probability of an independent activation of *post* location by emerging or other (spurious) sequences.

The increased probability of activation in the Starter motif effectively increased the length of the pathways, with more STAS being generated and propagated along the pathway (Fig. 4*A*, right panel). A mid in-degree region could also occur at the end of a pathway, and local changes there also had a similar effect of elongating the pathway. However, such regions did not change the global activity in the network in a measurable manner (not shown).

#### Repeat-Stop

A mid in-degree region may also occur along a pathway, i.e., between two high in-degree regions, featuring unreliable transmission of activity. A decrease in the local connectivity in such regions could essentially “stop” the transmission. Alternatively, an increase in the local connectivity would increase the reliability of the transmission. This is akin to a repeater device in electronic communication systems. Therefore, we refer to these locations as Repeat-Stop motif (Fig. 4*B*,*C*, left panel).

To illustrate the effect of changing the connectivity strength of the Repeat-Stop motif, we followed the same approach as was used above for the Starter motif (here, the *pre* spot is on the incoming pathway, and the *post* spot is on the outgoing pathway). As expected, increasing the excitability of the Repeat-Stop motif increased the number of sequences that traversed both detection spots *pre & post* indicating a higher transmission probability *pre* → *post* [i.e., *P*(*post*|*pre*): 64% (patch) vs 41% (baseline)]. Note that the overall sequence count at *pre* and *post* did not change much individually, and thus could not account for the increased STAS count of *pre & post* (Fig. 4*B*, middle and right panel). Similarly to the Starter motif, the activation of *post* was shifted toward STAS that already crossed the *pre* location [i.e., *P*(*pre*|*post*): 46% (patch) vs 35% (baseline)].

By contrast, a decrease in the local connectivity of the Repeat-Stop motif lowered the transmission probability as almost no sequences traversed both detection spots *pre & post* [i.e., *P*(*post*|*pre*): 4% (patch) vs 41% (baseline); Fig. 4*C*, right panel]. In addition, the average activation decreased far downstream along the pathway (Fig. 4*C*, middle panel).

#### Anti-Repeat

It is important to note that the specific location of modulation is crucial for the Repeat-Stop motif. For instance, when we instead increased the local connectivity in a high in-degree region just following a Repeat-Stop motif in a pathway, the previously detected “Repeat” pattern could no longer be observed as the transmission rate *pre* → *post* decreased [i.e., *P*(*post*|*pre*): 37% (patch) vs 41% (baseline); Fig. 4*D*]. Therefore, we refer to such regions as Anti-Repeat motifs. The increased excitability in the Anti-Repeat motifs evoked sequences independently, and these spurious sequences then suppressed the transmission *pre* → *post* temporarily due to the asymmetric shadowing inhibition (cf., Fig. 1*B*, inset). Hence, fewer sequences that were detected in *post* originated in *pre* [i.e., *P*(*pre*|*post*: 24% (patch) vs 35% (baseline); Fig. 4*D*, right panel].

### Mid in-degree regions alter the dynamics of branching and merging of sequences

Thus far, we have discussed motifs that affect a single pathway, affecting their transmission probability and length. Given the geometry of connectivity in our network, pathways merged and split. Here, sequences competed with each other due to the shadowing inhibition. A mid-degree region close to the branching point could bias the activity by modulating the competition or cooperation between pathways. Thus, new motifs such as Gate and Select arose in the network.

#### Select

**Figure 5.**
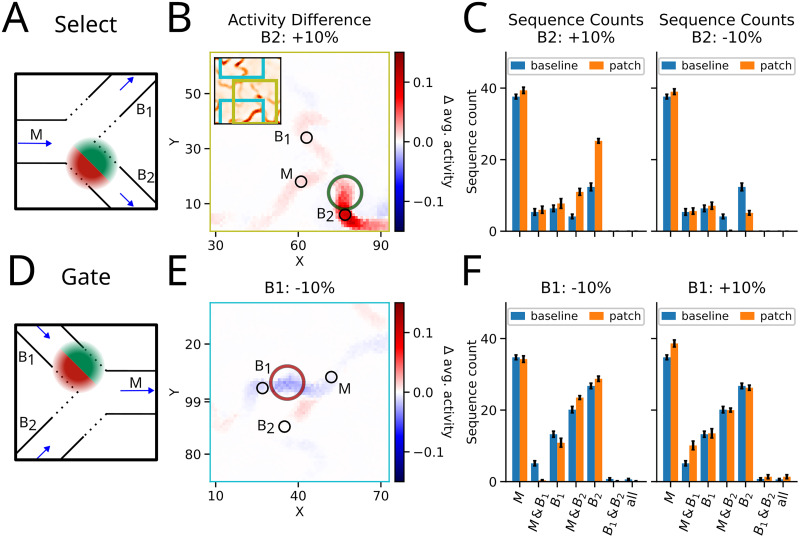
Effect of changes in the local connectivity at intersections. ***A***, Select motif: a change in the local connectivity of neurons in this region could change the probability of selecting one outgoing branch over the other. ***B***, Difference in average activity induced by local increase in the connectivity (green circle) and the baseline shown in the panel Figure 1*E* (see also Fig. 3*B*). The patch location is indicated with the green circle. For visual clarity, we zoomed into the motif location as indicated by the inset. ***C***, Sequence count registered across detection spots on the main pathways *M*, and the branches *B*_1_ and *B*_2_. Left panel: strengthening the local connectivity toward branch *B*_2_ increased the transmission rate toward branch *M* → *B*_2_ (see Methods). Right panel: weakening of the local connectivity toward branch *B*_2_ reduces transmission toward it. Sequences are almost unaffected toward branch *M* → *B*_1_. Error bars indicate SEM across simulations (*S*_seeds_ = 8). ***D***, Gate motif: local modulation on a branch alters the transmission probability toward the main branch. ***E***, As in ***B*** for the Gate motif, here the patch location is indicated with a red circle. ***F***, Sequence count registered across detection spots on the branches *B*_1_, and *B*_2_, and the main pathway *M*. Left panel: a reduction of local connectivity on branch *B*_1_ increases the transmission *B*_2_ → *M*. Right panel: an increase of local connectivity on *B*_1_ also resulted in elevated transmission *B*_1_ → *M*. Error across simulations (*S*_seeds_ = 8).

Consider a region in the network where one pathway (Main pathway *M*) diverges into two different pathways (Branches **B*_1_, *B*_2_*, Fig. 5*A*). Here, it would be advantageous to *select* the branch for further sequence propagation. When the activity from pathway *M* arrives at the branching point, three scenarios could occur: (1) the activity dies as transmission to either branch fails; (2) the activity is transmitted to one of the branches, or (3) both branches become activated. An increased synaptic strength in the Select motif (Fig. 5*A*, green patch) could bias the activity transmission along one branch (here symbolically to branch *B*_2_), which might also affect activity in the other branch (Fig. 5*B*).

For instance, modulation of the connectivity toward branch *B_2_* increased the transmission *M* → *B*_2_ [i.e., *P*(**B*_2_*|*M*): 29% (patch) vs 11% (baseline)] while the *M* → *B*_1_ transmission remained on a similar level [i.e., *P*(**B*_1_*|*M*): 16% (patch) vs 14% (baseline); Fig. 5*C*, left panel].

As expected, a reduction of local connectivity at the Select motif also changed the dynamics around the intersection. The transmission to the modulated branch *M* → *B*_2_ was essentially blocked [i.e., *P*(**B*_2_*|*M*): <1% (patch) vs 11% (baseline); Fig. 5*C*, right panel; Fig. S5*A*]. Although competition was reduced, the transmission to the unmodulated branch continued almost unchanged (*M* → *B*_1_ increased from 14% to 15%). Furthermore, the spontaneous emergence of STAS also changed (sequence count for only *B*_1_ or *B*_2_, respectively).

#### Gate

In a scenario where two pathways merged, the interaction between two incoming sequences could be modulated by varying the local connectivity in the pathways just before the merging point.

In the simplest case, local weakening of the connectivity of branch *B*_1_ essentially disconnected the *B*_1_ pathway, halting transmission from branch *B*_1_ → *M* (Fig. 5*D*–*F*). This effectively *gated* sequence progression, allowing transmission only from the branch *B*_2_ → *M*. Thus, due to the lack of competition of arriving sequences, *B*_2_ → *M* effectively acted as a single pathway (Fig. 5*E*). Consequently, the transmission from *B*_2_ → *M* was increased [i.e., *P*(*M*|*B*_2_): 82% (patch) vs 75% (baseline)], while transmission probability from *B*_1_ → *M* was abolished [i.e., *P*(*M*|*B*_1_): 3% (patch) vs 39% (baseline)], hence the name Gate motif (Fig. 5*F*, left panel).

However, if we strengthened local connectivity in branch *B*_1_, its effect on the transmission unfolded in two ways: in some cases, the elevated activity induced stronger competition between the STAS of *B*_1_ and *B*_2_. As a consequence, the transmission probability from *B*_1_ → *M* was increased [i.e., *P*(*M*|*B*_1_): 75% (patch) vs 39% (baseline)], while it remained on a similar level from *B*_2_ → *M* [i.e., *P*(*M*|*B*_2_): 76% (patch) vs 75% (baseline); Fig. 5*F*, right panel; Fig. S5*B*]. In rare instances, the two incoming STASs excited each other (cooperation) such that both sequences survived and merged despite the recurrent inhibition. Therefore, we also found activation of *all* detection spots: on average, only 1.375 sequences merged per simulation (4 s) compared to only 0.625 observed cooperative sequences in the baseline simulation (Fig. 5*F*, right panel).

**Figure 6.**
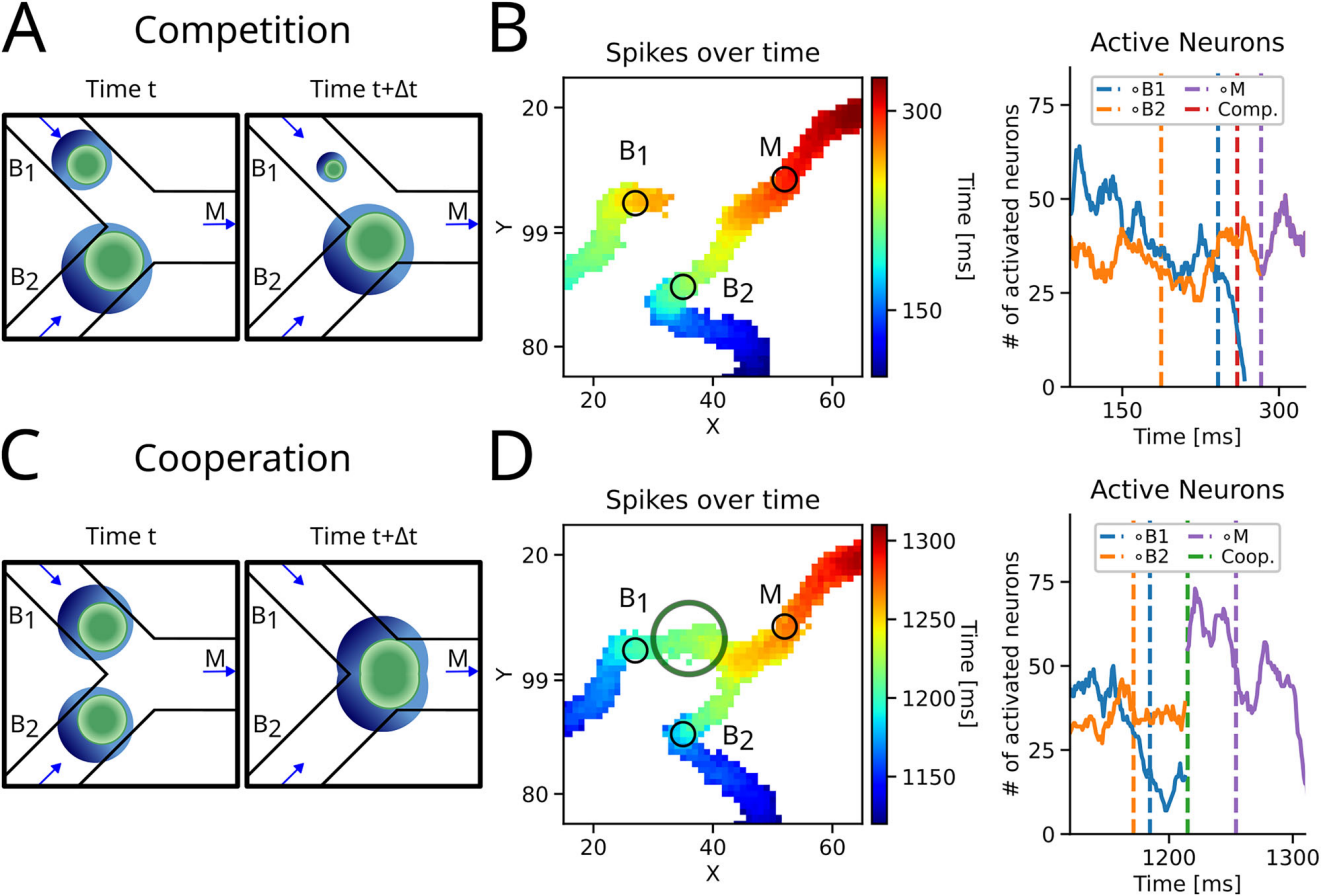
Competition and cooperation. ***A***, Schematic of competition: when the two incoming sequences are dissimilar in timing or strength, only one lasts, while the second fades. ***B***, Left panel: activity across space. Color indicate the time point of last activation. Note that the activity from branch *B*_2_ arrives earlier at the intersection than the activity from *B*_1_ (see also vertical orange and blue dashed lines in the right panel). Right panel: number of active neurons along each branch. Vertical dashed lines indicate the time that the sequences crossed the detection spot of the corresponding branch (at the spot *M* the orange line transforms to purple). The red dashed line indicates the time point of maximum competition (see Methods). ***C***, Schematic of cooperation: when two balanced sequences arrived simultaneously at the intersection, they cooperated and continued together on the main branch *M*. ***D***, Left panel: Same as in panel ***B*** but for cooperation. Note, the timing of the arriving sequences is similar, as well as the number of active neurons is balanced. Right panel: number of active neurons on each branch (*B*_1_ and *B*_2_) were similar, i.e., balanced before merging. The two sequences merged at *t* = 1,218 ms (transition from the orange and blue to the purple line; indicated by the dashed green line).

Whether competition or cooperation was emphasized depends on the relative strength and timing of the STASs. Given the connectivity in our network, activity traveling over the excitatory neurons (Fig. 6*A*,*C*, green region) created a trailing shadow of inhibition (Fig. 6*A*,*C*, blue region) whose strength depended on the size of the excitatory pool recruited by the sequence. Therefore, a strong STAS could destabilize or even suppress a weaker STAS (Fig. 6*B*). This phenomenon would be especially prominent when one sequence arrives earlier than the other (see crossing times on *B*_1_ and *B*_2_ being temporally gapped). This asymmetric suppression is also emphasized in the Anti-Repeat motif (see above).

By contrast, if two balanced STASs approached the intersection (almost) simultaneously, the activity could merge, and joint transmission from both branches was possible (Fig. 6*D*). Merging at an intersection requires recurrent excitation to overcome the surround inhibition, which is weakest at the front of each moving bump of activity. There is thus a window of opportunity for cooperation that depends on the size and relative timing of the two activity sequences. We note that in our networks, cooperation was observed far less frequently than competition.

#### Task-specific network modulation

**Figure 7.**
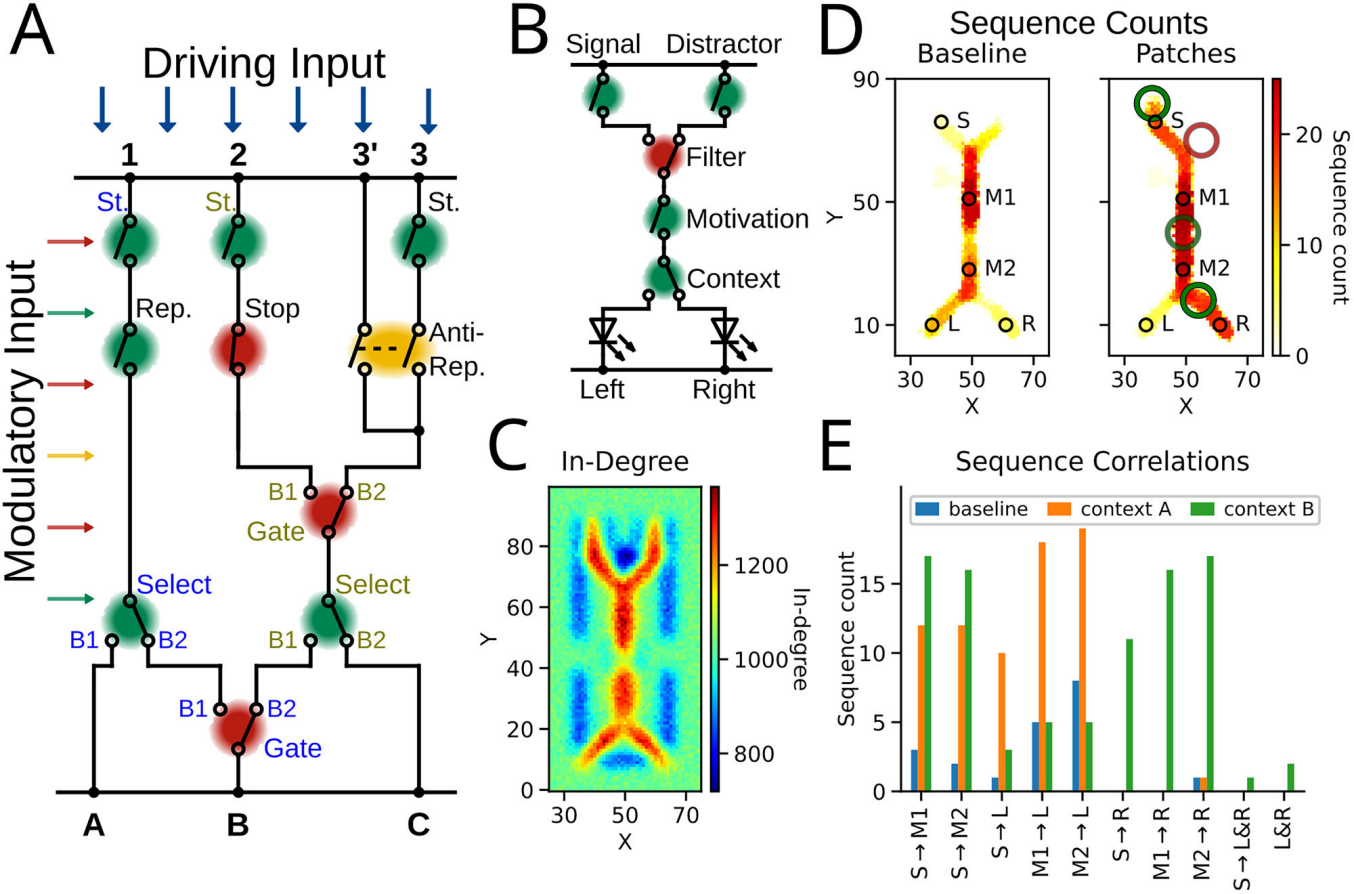
***A***, Schematic of the neural circuit. The driving input ignites sequences at the entry points 1, 2, 3, which can be modulated by the corresponding Start motif (abbreviated as St.). The modulatory input sets the switches, which results in various possible trajectories the sequence traverses. The read-out A, B, C can then be reached by sequences originated from multiple entry points. ***B***, Context-reversal task: Signal and Distractor are input signals. Several motifs represent task-relevant variables. The behavioral output was to choose either Left or Right (denoted by circuit representation of light-emitting diodes, LEDs). ***C***, In-degree of the network implementing the task in B. ***D***, Left panel: sequence counts on location in baseline condition. Right panel: sequence count on location with active motifs in context B (motifs as red and green circles, brightness indicates strength of the modulation) ***E***, Sequence correlation: indicates how many sequences were detected across the detection spots S, M1, M2, L, and R (spots are the black circles in ***D***). In the task condition (orange), more sequences are initiated and traverse the network more reliable than in the baseline (blue).

Thus far, we have unraveled the effects of motifs in isolation. Consequently, the next step is to observe the synergy among motifs in a task-specific context. Generally, interactions among motifs could arise in many flavors. In a more complex network, the input may initiate sequences at various starting points (for instance, Fig. 7*A*, #1, #2, or #3). Depending on how the “switches” (motifs of different kinds) are (de-)activated by modulatory input, a sequence could traverse the network and reach different endpoints or read-outs (here A, B, or C). In a large network with many pathways and multiple motifs simultaneously, sequences may start in one pathway and dynamically choose their trajectory in the network (Fig. 7). That is, a sequence originating from one pathway can arrive at various endpoints, but also one endpoint can be reached by many initial pathways.

We designed a context-reversal task to investigate how multiple motifs can interact and facilitate their effects (Fig. 7*B*). The task consists of two inputs, the signal and a distractor. Upon presentation of the signal, a context-dependent choice is required, while distractor trials require ignoring the input and withholding any response. In context A, the target output is an activation of *Left*. However, the same task could be performed in context B, in which the *Right* ought to be activated. In order to perform the task, the task-relevant features were implemented and represented as reliable and unreliable pathways to enable flexible control (Fig. 7*C*).

In the first step, a Start motif facilitated the initial progression of the incoming signal. Next, a Gate motif enacted a filter to remove the potential distractor. A Repeater motif could represent a higher-order signal, e.g., motivation. This could even be time-dependent and at some point, reverse to a Stop motif to lessen the participation in the task. Finally, the task-specific context is represented by a Select motif. Outside of the task, the branches that lead to *Left* and *Right* were by design symmetric (here, *Left* is only activated more often by the particular instantiation of the connectivity). However, context-dependent modulation could favor one branch over the other.

In the naive network, sequences arose in various locations of the network (Fig. 7*D*,*E*, baseline). We detected sequences in the signal pathway (S), at two points along the main path (M1, M2), and in the left (L) and right (R) choice branches. Additionally, some sequences originated in the signal pathway and traversed to the main branch (S→M1; S→M2), and even to the *Left* choice (Fig. 7*E*, S→ L; detection spots in *D*).

Considering context B with its target choice *Right*, modulation altered the network dynamics in a strong and systematic way. More sequences were initiated and transmitted with high reliability, even across the network, i.e., ∼65% of sequences that emerged from the signal pathway traversed to the right choice (Fig. 7*E*, S→R). Importantly, the correct output *Right* was activated frequently, while the wrong output *Left* was overall less active. Performing context-reversal, that is, responding appropriately when the environment is changed to context A, requires only the adaptation of the Select motif toward the left choice. As a result, more sequences traversed from the signal pathway to the target choice *Left* (Fig. 7*E*, S→L) with an overall transmission probability of ∼83%.

This example demonstrates how several motifs could work synergistically to perform a context-reversal task. In general, each motif could represent a task- or behavior-specific variable, and more complex tasks and features like redundancy could be implemented in larger networks (Fig. 7*A*).

## Discussion

To explain complex animal behavior, Donald Hebb introduced the idea of “Phase Sequences” (Hebb, 1949). Sherrington similarly had envisioned the brain as an “enchanted loom,” where “millions of flashing shuttles weave a dissolving pattern, always a meaningful pattern though never an abiding one; a shifting harmony of subpatterns” (Sherrington, 1940). These conceptual ideas require that neuronal networks in the brain can not only exhibit sequential activity but also rapidly switch and reconfigure the sequences. A number of mechanisms exist to explain the emergence of sequences (e.g., Hebbian plasticity, symmetry breaking due to synaptic depression or neuron adaptation, and asymmetric connectivity due to neuron morphologies). However, the problem of rapid switching among different sequences has received little attention. In some sense, this question of rapid switching relates to a question Larry Abbott posed: “Where are the switches on this thing?” (Abbott, 2006).

### Small switches with big leverage

In a network that is intrinsically poised to generate spatio-temporal sequences, “switches” are needed to control the magnitude (on/off/amplify) and direction of the activity. A solution to this problem is to control the magnitude (Vogels and Abbott, 2009) or timing of excitation and inhibition (Kremkow et al., 2010). Here we show that in networks with spatially correlated anisotropic connectivity of individual neurons (Spreizer et al., 2019) several locations naturally emerge that can guide sequential activity. A small (±10%) modulation of connectivity of as few as 40 neurons in these regions is sufficient to flexibly route sequential activity.

These observations suggest that stimulation of heterogeneous networks in the brain should be location-specific. In fact, there should be regions where activation/inactivation of neurons will elicit a rich network response, while other locations will display no visible impact on the network activity. The magnitude of the network response will, however, also depend on the strength of the modulation (Fig. 2*C*).

Moreover, spatially localized stimulation is more likely to generate a larger response than stimulation of neurons randomly distributed in the network. Note that here a large response implies that we should observe a large reshuffling of activity at the population level as sequential activity would be rerouted—recruiting new neurons and silencing others (Fig. 2). Thus, a local modulation of such “switches” could effectively alter the dimensionality and geometry of the manifold of neural activity.

### Where are the locations that have a big impact on network activity?

Neuron degree (in- or out-degree) in the brain is likely to be heterogeneous, given the neuronal chemical specificity and morphology (i.e., axonal and dendritic arbors are not isotropic in space). Furthermore, synaptic plasticity may also introduce additional heterogeneity in network connectivity. Thus, some neurons will have more connections than others. When the heterogeneity of neuron degree is uniformly distributed in the space, it may not have much impact (Spreizer et al., 2019). However, when there are regions of high, medium, or low degree, non-trivial activity dynamics emerge (Spreizer et al., 2019). From the perspective of controlling the activity dynamics, interactions among different degree regions play a crucial role.

Low in-degree neurons do not see much of the network activity, and therefore changing their connectivity strength by a small amount is not very effective in influencing the network dynamics. On the other hand, high in-degree regions already receive strong input from the network, and therefore small change in their connectivity also does not have much influence on the network activity. By contrast, medium in-degree regions are well poised to take advantage of the small modifications in connectivity/excitability and switch the network activity in a big way. The impact of a change in the connectivity/excitability of such neurons depends on the local neighborhood. Various motifs arise that can supervise sequence transmission and steer sequential activity at pathway intersections (Figs. 4, 5).

In our model, the spatial distribution of neuron in-degree determines their impact on the network dynamics. To some extent, a distribution of synaptic strengths can also do the same. Recently, Riquelme et al. (2023) showed that in a network, when synaptic weights are drawn from a log-normal distribution, feedforward pathways emerge where neurons are connected by strong synapses. In that model, feedforward pathways are linked by weaker connections, and stimulation of these links grants effective control of the sequential activity. Since connections in the mammalian cortex are relatively weaker, our mechanism may be more suited for the control of activity in the mammalian brain. Here, we have argued that certain medium in-degree neurons form the basis of high-impact locations. This is simply because in our model in-degree is variable. The argument should also hold when either out-degree or both in- and out-degree are heterogeneous and have a spatial distribution, but this remains to be shown.

### Targeting the high-impact locations

How could upstream networks target these neurons (spatial locations in the network) that have a high-impact on network activity? One possibility is that projections from an upstream network impinge in a spatially compact manner. This external input can directly drive the neurons closer to the threshold. This input, of course, has to be precisely timed with the arrival of a sequence to enable (or disable) further propagation. Moreover, such a stimulus could be strong enough to inject an additional sequence into the network.

Alternatively, neuron excitability or connectivity can be transiently modulated by NMs which are released in a spatially patchy manner (Patriarchi et al., 2018; Sun et al., 2018; Hamid et al., 2021; Glaeser-Khan et al., 2024). Neuromodulation-induced changes in the excitability or synaptic strength can occur on behaviorally relevant timescales (hundreds of ms). Neuromodulatory neurons typically have a rather large axonal arbor, and they can simultaneously target neurons with different in-degrees in a non-specific manner. Although many different neurons are modulated, the strongest effect on the dynamics may be manifested by affecting post-synaptic neurons with an overall mid or mid-to-high in-degree.

Finally, synaptic plasticity and learning could also modify the connectivity of incoming projections over longer timescales and bias it toward medium in-degree neurons. A correlation-based learning rule could unfold the synaptic weights in the following way: due to the high intrinsic activity of pathways, the correlation of input and activity would be low. On the other extreme, low in-degree regions are mostly silent and hence would not follow the input drive either. However, a medium in-degree region would be highly responsive to the input and thus form the strongest synapses. Consequently, inputs from an upstream network may be systematically potentiated if they impinge on medium in-degree neurons.

### What does it mean for computations in the brain?

Performing any task in a natural environment involves multiple steps. If each component of a task (e.g., extending the arm, opening the finger, etc.) is represented by sequential activity of neurons, then behavior should involve dynamic interactions among sequences. Cooperation and competition naturally arise, but strengthening, stopping, gating, and routing should be possible. The “switches” we have described can form an important component of neural circuitry that allows for dynamic control of sequences.

As an example, we illustrated how local modulation sets the switches in the desired positions to solve a context-reversal task. The key concept is that the switches represent task-relevant variables—filtering competing input, keeping the motivation high, and selecting the target output. It is unlikely that there exists isolated forms with well-defined inputs and outputs in the brain. More probable is that one finds a plethora of motifs in a network with many neurons. Assuming that inputs and outputs are redundantly distributed across the network, many pathways emerge from input to output crossing (several) motifs in a task-/context-dependent manner. It remains for future work to investigate how a task may be solved by exploiting the intrinsic sequential dynamics and connection heterogeneities which form the basis of “switches.”

Other properties of sequences, namely the number of neurons recruited at any given point in time and the propagation velocity, may also play a role and contribute to the computational power (Lehr et al., 2024, 2025). Although not investigated in this study, background activity or forms of short-term synaptic plasticity may regulate sequence dynamics and propagation velocity (Murray and Escola, 2017).

### Model predictions

Despite simplifications and assumptions (as needed by models), our model makes several testable predictions that could provide data to both support the model and extend it. First and foremost, stimulus response should depend on spatial locations. That is, local stimulation will evoke richer network responses compared to spatially distributed input, as shown in Figure 2. In terms of network connectivity, projections from an upstream network should be spatially compact and should target neurons that have mid-range in-degree in the local network.

Given the presence of “switches,” sequences traversing only parts of a pathway would be highly reliable, and the full sequence is finally composed of a few (or many) shorter elemental sequences. This perspective interprets trial-by-trial variability as a flexible web of computations.

In most brain regions, multiple NMs converge simultaneously in space and/or time. In our study, such convergence of antagonistic (i.e., one NM excites and the other inhibits) NMs on the same neurons can provide bidirectional control of the “switches.” In fact, regions where multiple NMs converge could be the “switches” of the network.

## Code Availability

The code for reproducing the simulations and the analyses can be found at GitHub https://github.com/Jiggaboy/local_modulation.git.
